# Characterization of a new member of *Iridoviridae*, Shrimp hemocyte iridescent virus (SHIV), found in white leg shrimp (*Litopenaeus vannamei*)

**DOI:** 10.1038/s41598-017-10738-8

**Published:** 2017-09-19

**Authors:** Liang Qiu, Meng-Meng Chen, Xiao-Yuan Wan, Chen Li, Qing-Li Zhang, Ruo-Yu Wang, Dong-Yuan Cheng, Xuan Dong, Bing Yang, Xiu-Hua Wang, Jian-Hai Xiang, Jie Huang

**Affiliations:** 1 Qingdao Key Laboratory of Mariculture Epidemiology and Biosecurity, Key Laboratory of Maricultural Organism Disease Control, Ministry of Agriculture, Function Laboratory for Marine Fisheries Science and Food Production Processes, Qingdao National Laboratory for Marine Science and Technology, Yellow Sea Fisheries Research Institute, Chinese Academy of Fishery Sciences, Qingdao, 266071 China; 2 Shanghai Ocean University, Shanghai, 201306 China; 3 Dalian Ocean University, Dalian, 116023 China; 4 Key Laboratory of Experimental Marine Biology, Institute of Oceanology, Chinese Academy of Sciences, Qingdao, 266071 China

## Abstract

A newly discovered iridescent virus that causes severe disease and high mortality in farmed *Litopenaeus vannamei* in Zhejiang, China, has been verified and temporarily specified as shrimp hemocyte iridescent virus (SHIV). Histopathological examination revealed basophilic inclusions and pyknosis in hematopoietic tissue and hemocytes in gills, hepatopancreas, periopods and muscle. Using viral metagenomics sequencing, we obtained partial sequences annotated as potential iridoviridae. Phylogenetic analyses using amino acid sequences of major capsid protein (MCP) and ATPase revealed that it is a new iridescent virus but does not belong to the five known genera of *Iridoviridae*. Transmission electron microscopy showed that the virus exhibited a typical icosahedral structure with a mean diameter of 158.6 ± 12.5 nm (n = 30)(v-v) and 143.6 ± 10.8 nm (n = 30)(f-f), and an 85.8 ± 6.0 nm (n = 30) nucleoid. Challenge tests of *L. vannamei* via intermuscular injection, per os and reverse gavage all exhibited 100% cumulative mortality rates. The *in situ* hybridization showed that hemopoietic tissue, gills, and hepatopancreatic sinus were the positively reacting tissues. Additionally, a specific nested PCR assay was developed. PCR results revealed that *L. vannamei*, *Fenneropenaeus chinensis*, and *Macrobrachium rosenbergii* were SHIV-positive, indicating a new threat existing in the shrimp farming industry in China.

## Introduction

*Iridoviridae* is a family of large icosahedral viruses with diameters in the range from 120 to 300 nm, which may even be up to 350 nm (e.g. genus *Lymphocystivirus*). The virion core contains a single linear double stranded DNA (dsDNA) molecule of 140–303 kbp, a value that includes both unique and terminally redundant sequences^1^. This family of viruses is characterized by its wide host spectrum, including invertebrates (such as insects), poikilothermic vertebrates, such as fish, amphibians and reptiles^1^. Based on their particle sizes, host range, DNA cross-hybridization, the presence of a methyltransferase, and the sequence of major capsid protein (MCP), family *Iridoviridae* is subclassified into five genera, including *Iridovirus, Chloriridovirus*, *Ranavirus, Lymphocystivirus*, and *Megalocytivirus* ^1^. Viruses of the genera *Iridovirus* and *Chloriridovirus* primarily infect insects while the species of genera *Megalocytivirus* and *Lymphocystivirus* are associated with fish hosts. Viruses of genus *Ranavirus* are known to cause disease in amphibians, reptiles, and finfish^1–3^. Furthermore, it was reported that some viruses of *Iridoviridae*, such as a Irido-like virus in marine crab *Macropipus depurator*, could infect crustaceans^4^, Sergestid iridovirus (SIV) in sergestid shrimp *Acetes erythraeus* ^5^, a putative iridovirus in penaeid shrimp *Protrachypene precipua* ^6^, and invertebrate iridescent virus 31 (IIV-31) in pill bug *Armadillidium vulgare* ^7^. Recently, an iridescent virus was identified from freshwater lobster *Cherax quadricarinatus* and named Cherax quadricarinatus iridovirus (CQIV)^8^.

White leg shrimp *Litopenaeus vannamei* is one of the most important crustacean species in worldwide aquaculture, especially for the coastal developing countries. The production of this species has accounted for 53.1% of total production of crustaceans for the world aquaculture and 98.6% of *L. vannamei* was produced in developing countries in 2014^9^. However, emerging diseases recently occurred in farmed *L. vannamei* have significantly and negatively impacted shrimp farming industry along with the spread of the species^10^.

Samples of *L. vannamei* were collected from a shrimp farm where massive die-offs occurred in Zhejiang Province, China, in 2014 during our epidemiological investigation. In this study, we analyzed the diseased shrimp samples using viral metagenomics sequencing. Based on the sequenced data, we identified and described a novel virus of family *Iridoviridae*, which was temporarily specified as shrimp hemocyte iridescent virus (SHIV).

## Results

### Observation and detection of diseased shrimp

The samples of *L*. *vannamei* (No. 20141215) collected from the pond with massive die-offs exhibited obvious clinical signs, including empty stomach and guts, pale hepatopancreas, and soft shell. The shrimp samples were tested and demonstrated to be free of white spot syndrome virus (WSSV), yellow head virus (YHV), Taura syndrome virus (TSV), infectious hypodemal and hematopoietic necrosis virus (IHHNV) and Vibrio parahaemolyticus, which cause acute hepatopancreas necrosis disease (VPAHPND) by PCR or RT-PCR methods recommended by the World Organization for Animal Health^11^ and Flegel & Lo^12^.

### Histopathology

**Figure 1:**
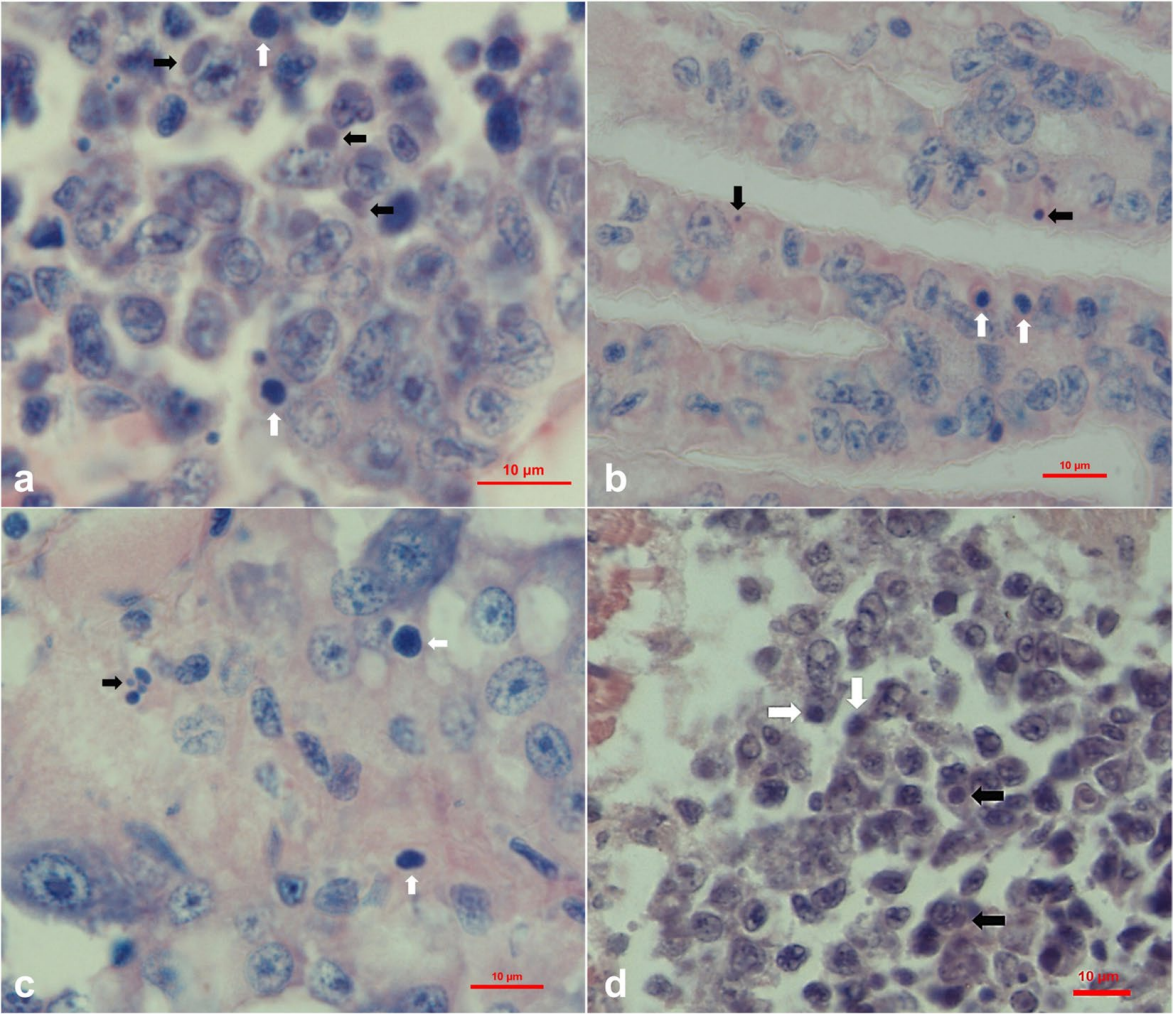
Histopathological features of Davidson’s alcohol-formalin-acetic acid (AFA) fixed *L. vannamei* in sample 20141215 (**a**,**c**,**e** and **d**). Black arrows show the basophilic inclusions while white arrows show the karyopyknotic nuclei. (**a**) Haematoxylin and eosin (H&E) staining of the hematopoietic tissue; (**b**) H&E staining of gills; (**c**) H&E staining of the sinus in hepatopancreas, and (**d**) H&E staining of periopods. Bar, 10 μm.

Cephalothoraxes of sample 20141215 were fixed and paraffin sections were prepared and stained with Haematoxylin and Eosin (H&E) staining. Histological examination showed that basophilic inclusions and karyopyknosis existed in hematopoietic tissue and hemocytes in gills, hepatopancreas and periopods (Fig. 1a–d).

### Sequencing and phylogenetic analyses

**Figure 2:**
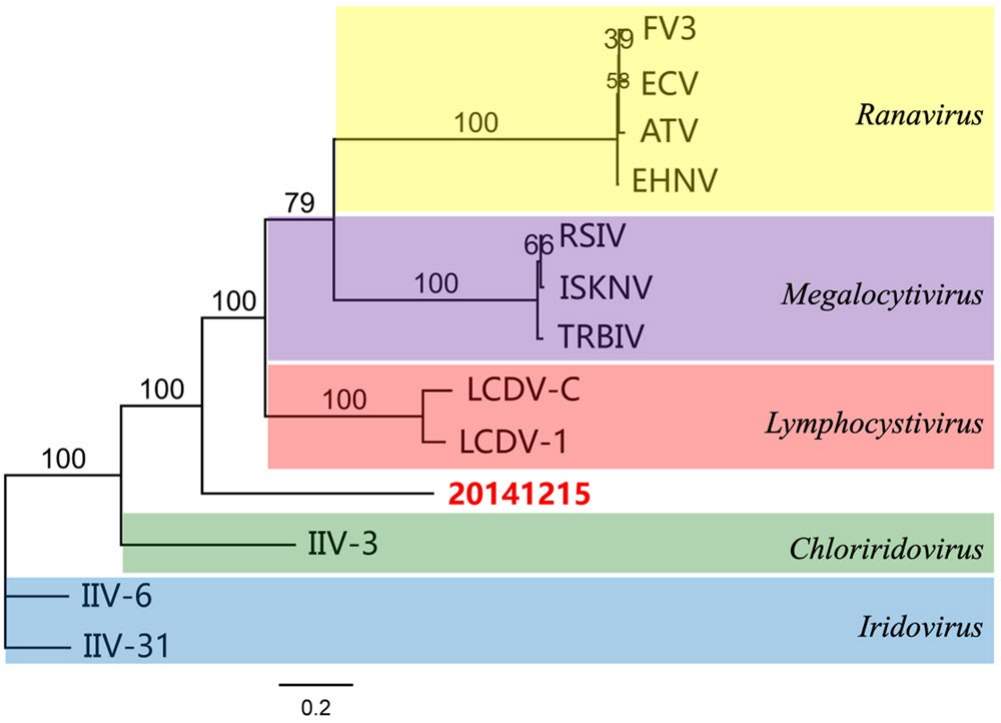
Phylogenetic tree based on the deduced amino acid sequences of shrimp hemocyte irido virus (SHIV) clone with MCP and ATPase sequences from other members of Iridoviridae (for virus abbreviations, see Tables 1 and 2). The tree was reconstructed by the maximum-likelihood method using Geneious 9.1.4 and the numbers indicate percentages of bootstrap support from replicates. Percentages of bootstrap values (1000 replicates) are shown. Bar, 0. 2 expected nucleotide substitutions per site.

A total of 1,492,644 sequence reads in 125 nt showed identity to the sequence from the family *Iridoviridae*. It was strongly suggested that a potential iridescent virus existed in the sample 20141215. Sequence assembly and BLAST search showed that the nucleic acid sequence of a 1434 bp fragment (GenBank access KY681039) shared 46%, 46%, and 45% identities in the amino acid sequences with those of the major capsid protein (MCP) from *Armadillidium vulgare* iridescent virus (AVIV), Invertebrate iridescent virus 6 (IIV6) and lymphocystis disease virus 1 (LDV-1), respectively, and that the amino acid sequence of a 1200 bp fragment (GenBank access KY681040) shared 52%, 51%, and 51% identities in the amino acid sequence with those of the ATPase from Lymphocystis disease virus 1 disease virus 1 (LDV-1), Epizootic haematopoietic necrosis virus (EHNV) and Lymphocystis disease virus-isolate China (LDV-C), respectively (Table 1). These BLAST results together with the assembled sequences provided additional supports to the possibility of a potentially new iridescent virus in the sample 20141215. To determine the phylogenetic relationship of this virus found in the sample 20141215 to other iridescent virus, we used amino acid sequences of MCP and ATPase to conduct alignments with those of other iridescent virus from GenBank. In the tree, the iridescent viruses used in the multiple alignments were subdivided into five groups: *Ranavirus, Megalocytivirus, Lymphocystivirus, Chloriridovirus* and *Iridovirus*. The potentially new iridescent virus 20141215 is located in a new branch which does not belong to any branch of the five genera (Fig. 2). These results of phylogenetic analyses of MCP and ATPase indicated that the 20141215 did not belong to any of the five classified genera of *Iridoviridae*.

**Table 1:** Percentage similarity of the amino acid sequences of MCP and ATPase of SHIV as compared with those of other members of *Iridoviridae*.

**Virus**	**MCP**		**ATPase**	
	**GenBank accession number**	**Sequence similarity to SHIV (%)**	**GenBank accession number**	**Sequence similarity to SHIV (%)**
***Ranavirus***				
FV3	ABI58271.1	38	AHM26101.1	43
ECV	YP_006347613.1	38	YP_006347705.1	43
ATV	ALN36406.1	38	YP_003852.1	43
EHNV	YP_009182013.1	38	YP_009182084.1	51
***Megalocytivirus***				
RSIV	BAC66968.1	39	BAK14298.1	44
ISKNV	ADU25248.1	39	NP_612331.1	45
TRBIV	AAT01301.2	38	ADE34443.1	44
***Lymphocystivirus***				
LCDV-C	YP_025102.1	43	YP_073585.1	51
LCDV-1	BAF57229.1	45	AAX54510.1	52
***Chloriridovirus***				
IIV-3	YP_654586.1	43	YP_654693.1	43
***Iridovirus***				
IIV-6	NP_149737.1	46	NP_149647.1	41
IIV-31	YP_009046748.1	46	YP_009046717.1	41
SIV	ABR37646.1	56	—	—

Note: The full names of the abbreviated virus names are as follows. FV3: Frog virus 3; ECV: European catfish virus; ATV: Ambystoma tigrinum virus; EHNV: Epizootic haematopoietic necrosis virus; RSIV: Red seabream iridovirus; ISKNV: Infectious spleen and kidney necrosis virus; TRBIV: Turbot reddish body iridovirus; LDV-C: Lymphocystisdisease virus-isolate China; IIV-3: Invertebrate iridescent virus 3; IIV-6: Invertebrate iridescent virus 6; IIV-31: Invertebrate iridescent virus 31; and SIV: Sergestid iridovirus.

### Confirmation of the causative pathogen by experimental challenge

**Figure 3:**
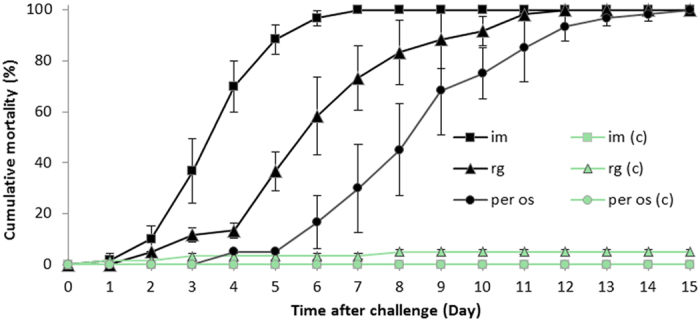
Cumulative mortalities of *L. vannamei* in experimental infections. Two groups of shrimp were challenged with filtrate of tissue extracts by either intermuscular injection (im) injection or anal reverse garvage (rg). Another group of shrimp was challenged via per os infection (per os). The the control groups were treated in the same way with PPB-His buffer in group im (c) and group rg (c), respectively, or fed with shrimp feed in group *per os* (c). Cumulative mortalities are shown as means of data from 3 replicates for each experimental group (each replicate included 30 individuals).

To further identify whether the potential iridescent virus was the aetiological agent, we conducted experimental challenges to two groups of health shrimp *L. vannamei* by either intramuscular (im) injection or anal reverse gavage (rg) with 0.22 µm filtered suspension of the pellet under 30% (w/w) sucrose spun from homogenate of the cephalothorax of frozen shrimp of sample 20141215. Oral administration (*per os*) with tissue of frozen shrimp of sample 20141215 was simultaneously conducted to another group of health shrimp *L. vannamei*. Results of challenge tests showed that cumulative mortality of shrimp in inter-muscular injection group (im), anal reverse garvage group (rg), and the oral administration group (*per os)* groups all reached 100% within two weeks of post-infection. The median lethal time (LT_50_) of the im group was 3.34 ± 0.32 d, which showed the fastest mortality. The LT_50_ of rg was 5.69 ± 0.48 d. The LT_50_ of per os was 8.11 ± 0.81 d. In contrast, there were less than 0–10% mortality of shrimp in the control group by im and rg with PPB-His buffer (376.07 mM NaCl, 6.32 mM K_2_SO_4_, 6.4 mM MgSO_4_, 14.41 mM CaCl_2_, and 26.10 mM histidine hydrochloride, pH 6.5)^23^ (Fig. 3).

**Figure 4:**
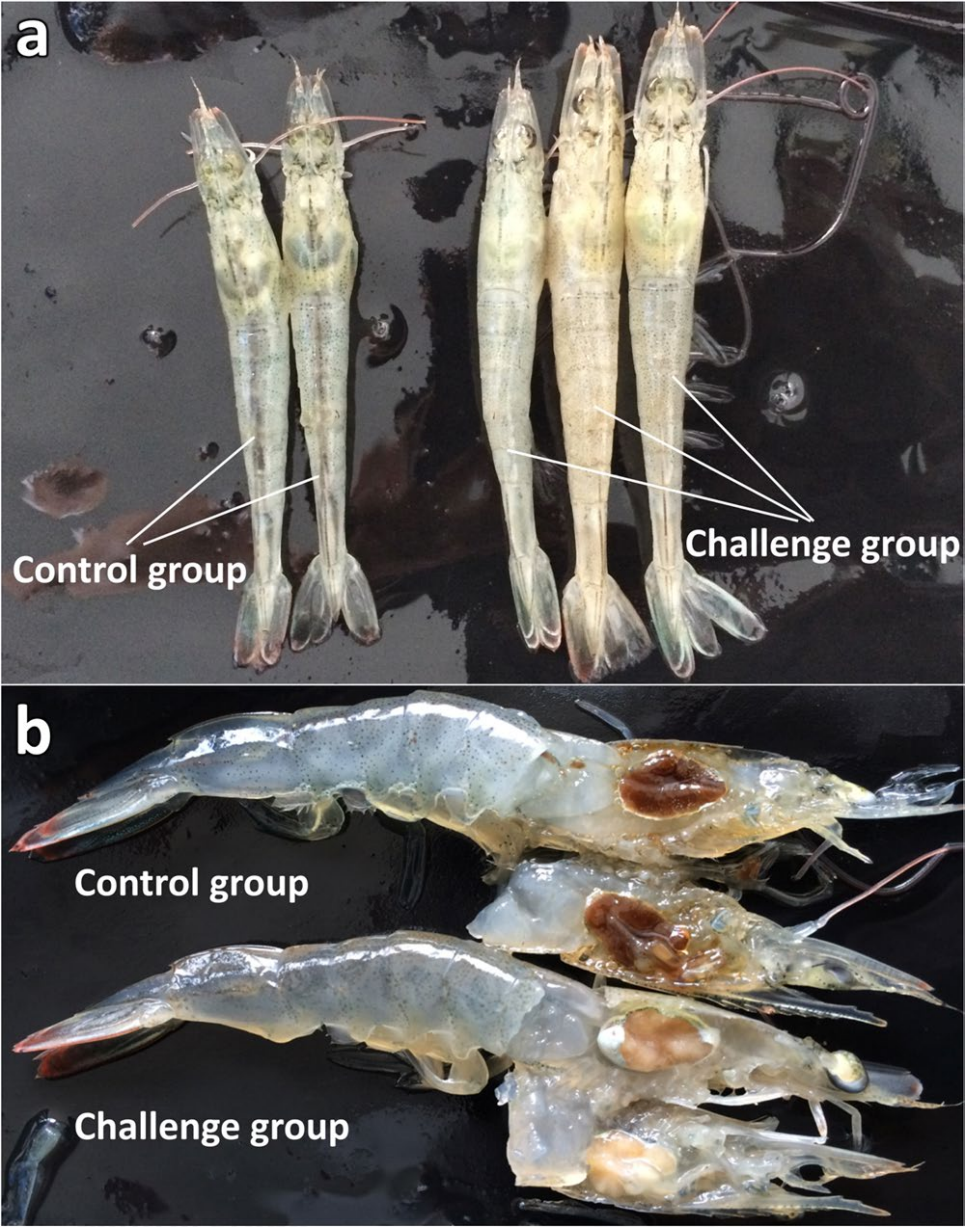
Clinical symptoms of *L. vannamei* challenged with the potential iridescent virus compared with those of the control group. (**a**) External appearance of the shrimp. (**b**) Section of hepatopancreas.

*L. vannamei* challenged with the viral preparation from the sample 20141215 exhibited the symptoms similar to those in the original individual sample, including empty stomach and guts in all diseased shrimp, slight loss of color on the surface and section of hepatopancreas, and soft shell in partially infected shrimp. One third of individuals had slightly reddish body (Fig. 4a,b). The moribund shrimp lost their swimming ability and sank to the bottom of water. The symptom and mortality were observed in the infected *L. vannamei* from post larvae to sub-adult shrimp in the laboratory.

Histopathological examination showed the presence of karyopyknosis and basophilic inclusions in hematopoietic tissue, hemocytes in gills and sinus of hepatopancreas. All of these observed symptoms were identical to those in the sample 20141215 collected from the diseased pond, which was in accord with the Rivers’ postulate. In addition, the results of RT-PCR and PCR analyses also indicated that all the shrimps from the infected group were strongly positive for the potentially new iridescent virus but no WSSV, IHHNV, VP_AHPND_, YHV and TSV were detected whereas all the above-mentioned pathogens were not detected in shrimps from the control group.

### Transmission electron microscopy (TEM) of ultrathin sections

**Figure 5:**
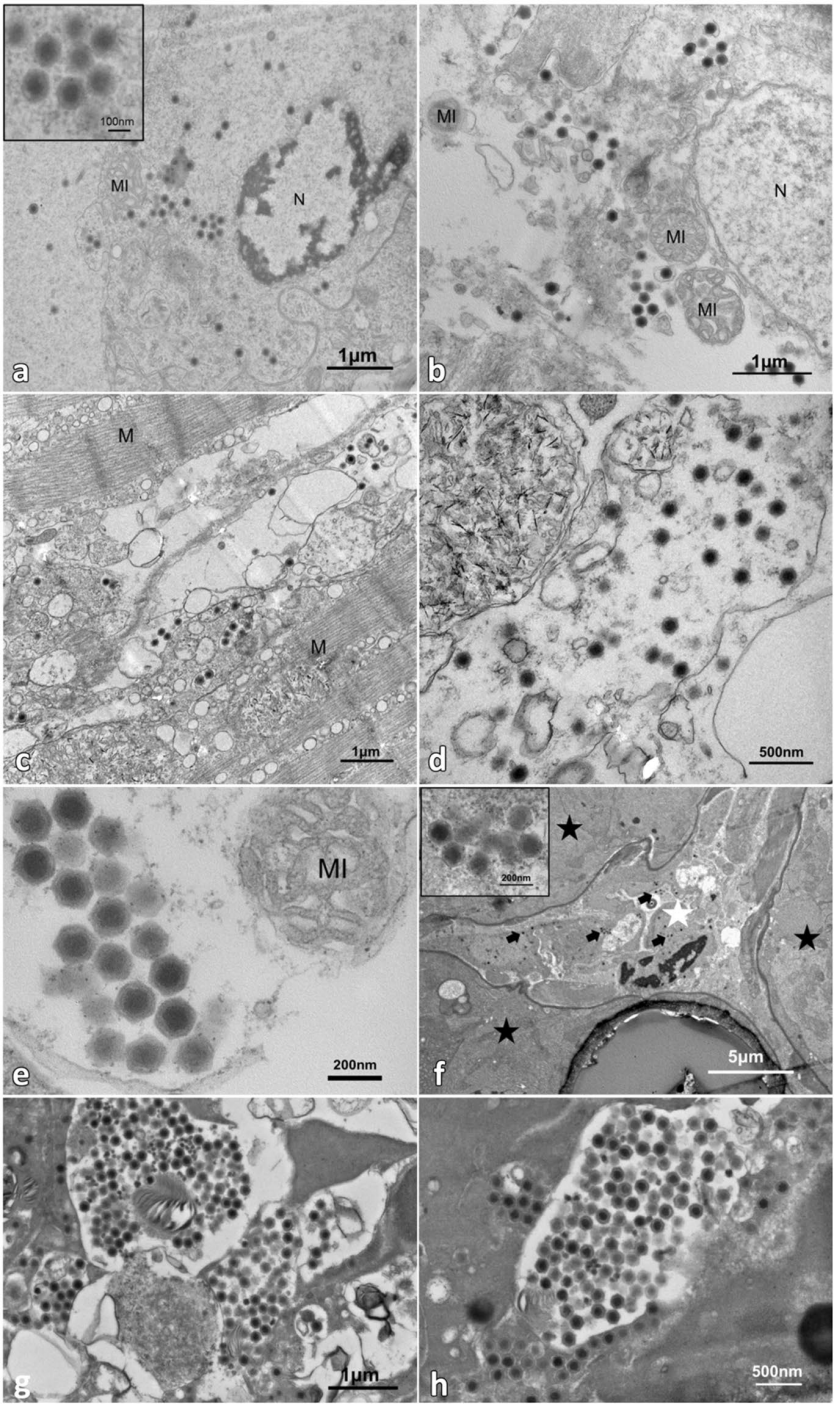
Transmission electron microscopy (TEM) of naturally infected *L. vannamei* showing a large numbers of virions in hemal sinuses of hepatopancreas (**a** and **b**) and skeletal muscle (**c** and **d**). TEM of the infected *L. vannamei* showing a large number of virions in the cytoplasm of hemocytes (**a**–**d**). MI: mitochondria; N: nucleus; and M: muscle; Black star: hepatopancreas tissue; and white star: hemal sinuses.

Visualization under TEM of ultrathin sections of the sample revealed the presence of enveloped icosahedral virus-like particles with typical non-enveloped iridescent viral structure in hemocytes of hemal sinuses in hepatopancreas and muscle (Fig. 5a–d). Non-enveloped virions were 158.6 ± 12.5 nm (n = 30) vertex to vertex (v-v) and 143.6 ± 10.8 nm (n = 30) from face to face (f-f), with capsomers layer (9.0 ± 1.5, n = 17) nm in thick), inner membrane (13.1 ± 3.8, n = 17) nm in thick) inside of the capsid, and a nucleoid [85.8 ± 6.0 nm, n = 30)]. TEM of *L. vannamei* challenged with the extracted supernatant from the sample 20141215 demonstrated the presence of the non-enveloped icosahedral virus-like particles with the same morphological characteristics (Fig. 5a–d). The virus particles (indicated by black arrow) existed in the cytoplasm of hemocytes of hemal sinuses (white star) of hepatopancreas and no virus-like particles were observed in the tubular epithelium of hepatopancreas (indicated by stars) (Fig. 5f). Virogenic stroma in the cytoplasm of hemocytes contained concentrated mature viral nucleocapsids with high electron density of nucleoid. Some incomplete virus-like particles in the same size with lower electron density nucleoid and small particles around 59.6 ± 6.8 nm (n = 15) with high electron density might be the assembled intermediates, indicating that the virus may be assembled in this area (Fig. 5g,h). The morphological characteristics of virions and virogenic stroma in cytoplasm observed under TEM are consistent with the characteristics of *Iridoviridae*.

### Virion purification

**Figure 6:**
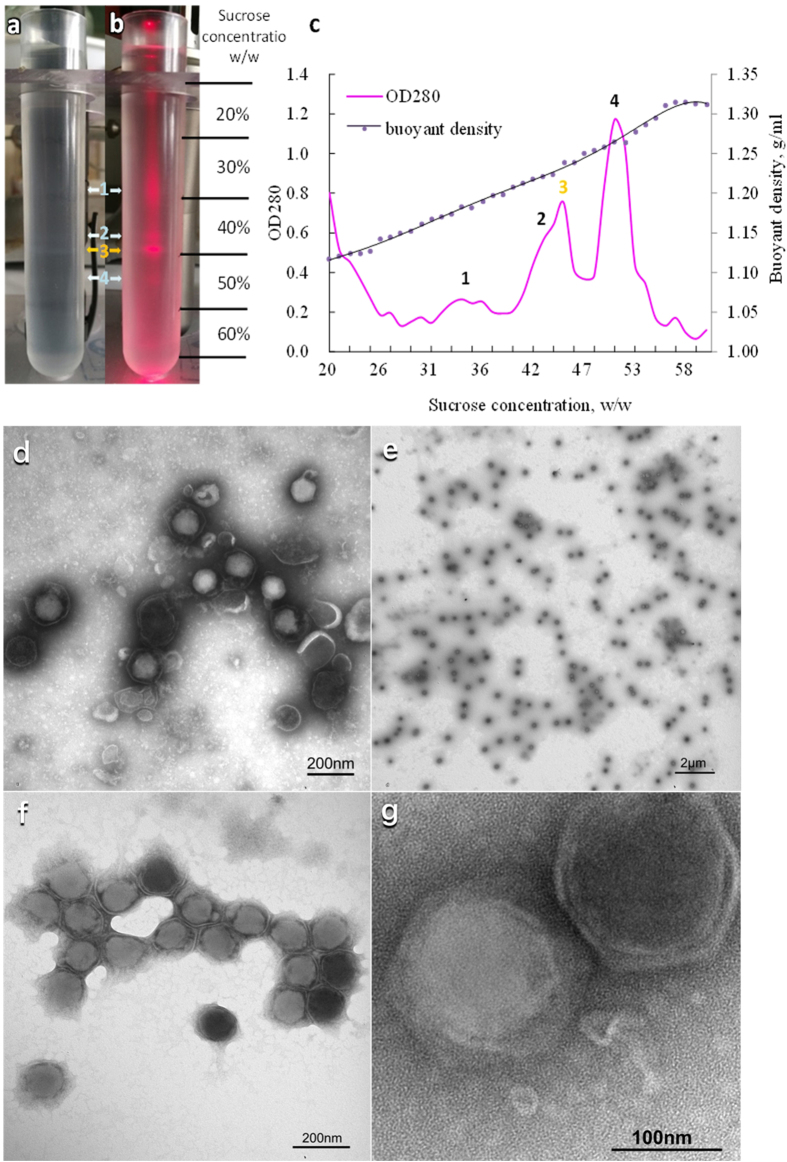
Centrifuged sucrose gradients of the new iridescent virus in natural light (**a**) and in red laser beam (**b**); Fractions of the gradients were taken and measured for buoyant density (pink) and the optical density at 280 nm (dark purple), (**c**). Four bands were pointed by the arrows and the 3^rd^ band was virions. TEM visualization of the negatively stained purification of the new iridescent virus. (**d**) Virions pelleted through a 30% (w/w) sucrose solution; (**e**), (**f**), and (**g**) Virions purified in the sucrose gradients.

After sucrose density gradient centrifugation, two cloudy bands with slightly blue color showed up in sucrose gradients under the original 40% (w/w) sucrose fraction (Fig. 6a). They were more observable with a laser irradiation (Fig. 6b). The major band had an OD_280_ of 0.752 and a buoyant density at 1.231 ± 0.01 g/mL (Fig. 6c), which was slightly lower than the equilibrium buoyant density of the virus. Visualization under TEM of the negatively stained grid with a drop from the band showed enveloped icosahedral viral particles with diameters of 160.2 ± 7.0 nm (n = 10)(v-v) and 142.6 ± 4.0 nm (n = 10) (f-f) (Fig. 6d–g).

According to the above results, a potentially new iridescent virus found in diseased farming shrimp *L. vannamei* is regarded as a naturally etiological agent of *L. vannamei*. Comparison of the morphological, physiological and phylogenetic characteristics of strain 20141215 with those of the species of the genera *Iridovirus*, *Chloriridovirus*, *Ranavirus*, *Lymphocystivirus*, and *Megalocytivirus* supported the conclusion that the isolate represents a novel species of a new genus. Hence, using shrimp hemocyte iridescent virus (SHIV), we temporarily specified the new virus in this paper and suggested to establish a new genus of *Iridoviridae, Xia’irido’virus*, which means *shrimp* (Xia is the Chinese phonetic of shrimp) iridescent virus.

### *In situ* hybridization (ISH) of SHIV

**Figure 7:**
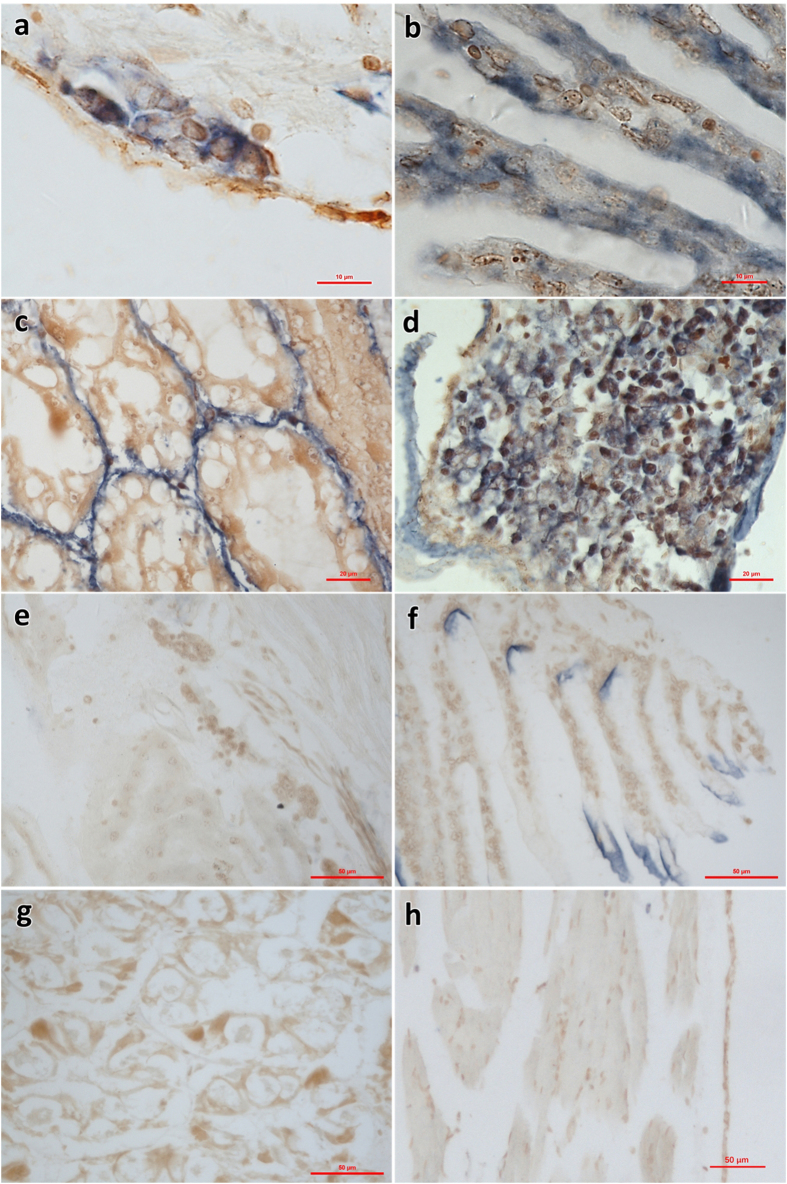
ISH using a digoxigenin labeled 279 bp probe for the shrimp hemocyte iridescent virus on histological sections of *L. vannamei*. (**a**)–(**d**) hematopoietic tissue, gills, hepatopancreas and periopods in SHIV-positive sample, respectively; (**e**)–(**h**) hematopoietic tissue, gills, hepatopancreas and periopods in SHIV-negative sample, respectively. In (**a**)–(**d**), blue signals were observed in the cytoplasm of the hemocytes of hematopoietic tissue, gills, sinus of hepatopancreas and periopods. In (**e**)–(**h**), no hybridization signal was seen on the same tissues of SHIV-negative *L. vannamei* except some non-specific signals on cuticle. Bar, 10 μm (**a** and **b**), 20 μm (**c** and **d**), and 50 μm (**e**–**h**), respectively.

A pair of primers targeting MCP gene of SHIV was designed to produce a digoxigenin labeled 279 bp amplicon by PCR. The labeled PCR product was used as the probe for ISH on thin sections of infected *L. vannamei*. Positive signals of ISH were observed in hematopoietic tissue and hemocytes in gills, hepatopancreas and periopods (Fig. 7a–d). However, the probe did not react with cells other than hemocytes and hematopoietic cells of infected shrimp, such as myocytes, cuticular epithelial cells, hepatopancritic tubular cells, and neurocytes etc. No positively hybridizing signal on the cuticles was observed in sections of the same tissues prepared from uninfected shrimp except some nonspecific signals on cuticle (Fig. 7e–h).

### Nested PCR assay for SHIV

**Figure 8:**
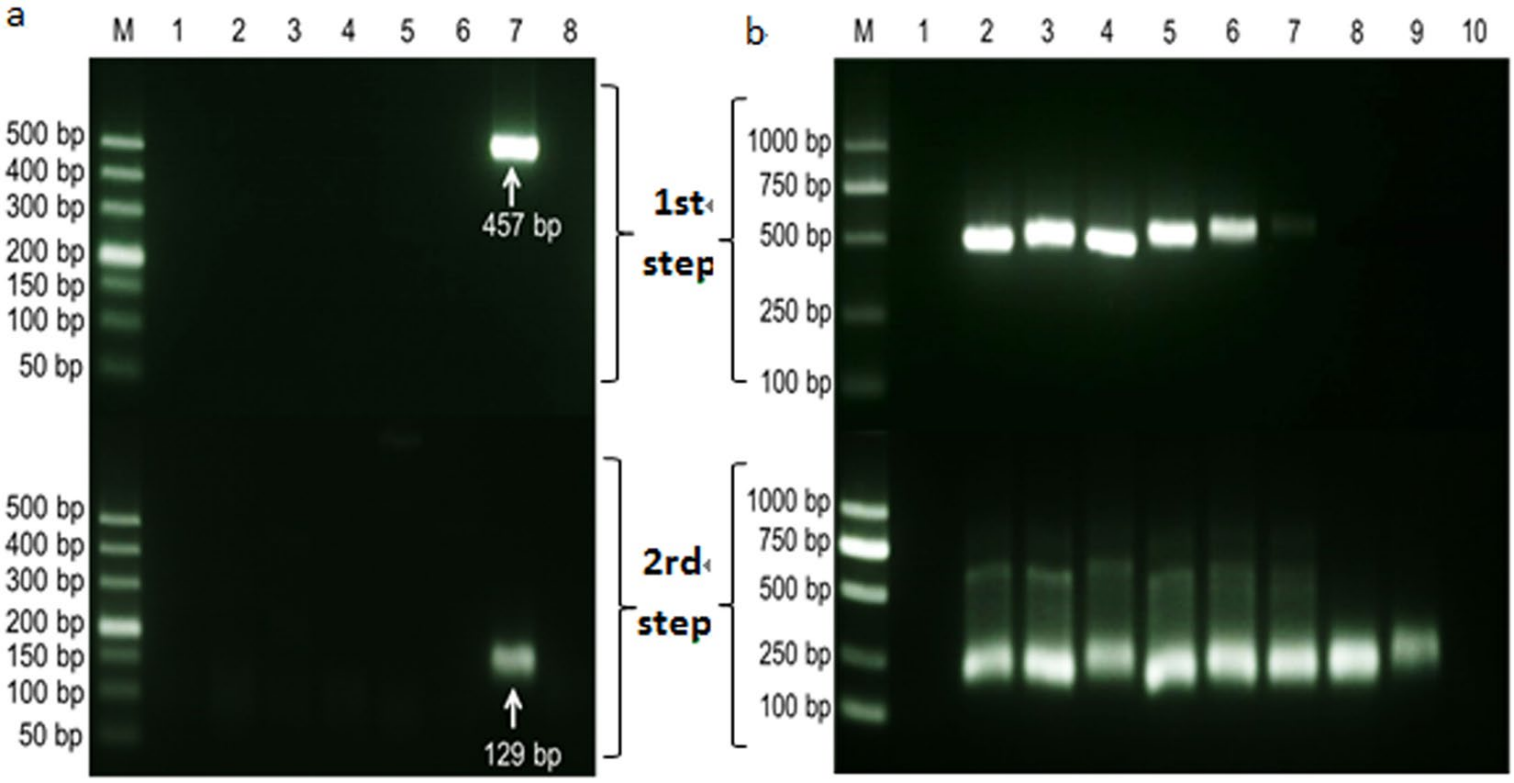
Detection of the shrimp hemocyte iridescent virus with the nested PCR method. (**a**) Specificity analysis of the nested PCR method. M, DL500 molecular mass marker; Lanes 1–6: PCR amplified products with DNA template samples extracted from health *L. vannamei* and *L. vannamei* infected with WSSV, IHHNV, HPV, AHPND, and EHP, respectively; lane 7: PCR amplified products with DNA template sample extracted from shrimp infected with the iridescent virus; and lane 8: negative control. (**b**) Sensitivity test of the nested PCR method. M, DL1000 molecular mass marker; Lane 1: negative control; Lines 2–10: PCR amplified products with the diluted DNA solutions (10^0^–10^−8^) of *L. vannamei* naturally infected with the shrimp iridescent virus.

A nested PCR assay was developed for highly sensitive detection of SHIV. The amplification result showed that the first step of the PCR produced a 457 bp amplicon and the second step of the PCR produced a 129 bp amplicon. Specificity analysis against the DNA samples extracted from shrimp infected with WSSV, IHHNV, HPV, VP_AHPND_ and EHP indicated that the nested PCR was specific for SHIV (Fig. 8a). Sensitivity test revealed that this nested PCR assay could detect DNA extracted from cephalothorax of SHIV-infected shrimp at 10° to 10^−7^ dilutions (Fig. 8b) and the limit of detection (LOD) was 36 fg DNA extracted from the infected tissue.

### Prevalence and distribution of SHIV

A total of 625 farmed shrimp, including 575 individuals of *L. vannamei*, 33 individuals of *F. chinensis*, 10 individuals of *Mb. rosenbergii*, and 7 individuals of *Mp. japonicas* were tested. Results showed that 15.8% (99/625) of these samples were SHIV-positives and the SHIV-positive rates in different species were 15.5, 15.2, 50.0 and 0.0 (%), respectively (Table 2). Relatively high positive rates of SHIV were detected in some coastal areas, including Zhejiang, Guangdong and Hebei Provinces.

**Table 2:** Shrimp species detected with the nested PCR specific for SHIV.

**Shrimp species**	**Negative**	**Positive**	**Total**	**Positive rate**
*L. vannamei*	486	89	575	15.5%
*F. chinensis*	28	5	33	15.2%
*Mb. rosenbergii*	5	5	10	50.0%
*Mp. japonicas*	7	0	7	0.0%
Total	526	99	625	15.8%

## Discussion

In recent years, epizooties of infectious diseases in farmed *L. vannamei* have caused massive economic losses in China. Diseased shrimps in many cases exhibited hepatopancreatic atrophy with fading color and a high mortality. It is commonly believed that acute hepatopancreatic necrosis of juveniles shrimp is related to AHPND caused by the virulent *Vibrio parahaemolyticus* ^14–17^.

In 2014, pond-farmed *L*. *vannamei* (2–3 cm) with massive die-offs exhibited hepatopancreatic atrophy with fading color, empty stomach and guts, and soft shell in Zhejiang Province, China. Nested PCR or RT-PCR results showed that it was negative for WSSV, IHHNV, VPAHPND, YHV and TSV^11^. Histological sections revealed that the disease might be caused by a suspected viral infection with obvious pathogenic symptoms, which did not match the histopathologic characteristics of infection with any known viruses and AHPND. High-throughput sequencing and BLAST revealed that a potential iridescent virus was present in the sample 20141215. The highest percentages of identities in amino acid sequence of the MCP and ATPase genes of the iridescent virus with those of the known members of family *Iridoviridae* were only 56% and 52%, respectively. The phylogenetic analysis of the two proteins support that SHIV does not belong to any known genus of family *Iridoviridae*. The challenge tests via invasive mode of intramuscular injection or noninvasive mode mimic natural methods, including reverse gavage or *per os* infection that could successfully pass filterable infectious agent from the disease shrimp to healthy *L*. *vannamei*, caused similar gross clinical symptoms and pathological changes. It has been proved that this virus is the causative agent of the disease. ISH with the digoxin labeled probe showed consistent signals with the inclusions observed in the H&E-stained histopathology in the hematopoietic tissue, gills, hepatopancreas and periopods. These results fulfilled Rivers’ postulates for the demonstration of a viral aetiology^18^. After having compared the morphological, physiological and phylogenetic characteristics of strain 20141215 with those of other iridescent viruses, we temporarily specified the aetiological agent as shrimp hemocyte iridescent virus (SHIV), which could cause shrimp hemocyte iridescent virus disease (SHIVD). A new genus of *Iridoviridae* was suggested as *Xiairidovirus*, which means *shrimp* iridescent virus.

To date, only 5 iridoscent viruses found in crustacean, including SIV infecting sergestid shrimp *Acetes erythraeus* were reported^5^: CQIV infecting freshwater lobster *Cherax quadricarinatus* ^8^, an irido-like virus infecting marine crab *Macropipus depurator* ^4^, a putative iridovirus infecting penaeid shrimp *Protrachypene precipua* ^6^, and IIV-31 infecting pill bug *Armadillidium vulgare* ^7^. Only completed genome sequence of IIV-31, gene *mcp* sequence of SIV, and gene *mcp* partial sequence of CQIV were published. It is noticed that CQIV can cause high mortality to *L. vannamei* in the experimental challenge. However, the test of challenge used an intramuscular injection method rather than a non-invasive experimental procedure. According to the OIE criteria for listing species as susceptible to infection with a specific pathogen^19^, *L. vannmei* cannot be confirmed as a susceptible host of CQIV based on currently published data. Therefore, we suppose that SHIV is the only reported iridescent virus with the MCP and ATPase sequences available and a susceptible host penaeid shrimp *L. vannamei*.

The clinical symptoms of SHIV infection include slight loss of color on the surface and cut of hepatopancreas, empty stomach and guts, soft shell in partially infected shrimp and slightly reddish body in one third of individuals. These symptoms are not similar to those caused by the infection with putative iridovirus in penaeid shrimp *Protrachypene precipua* with a whitish coloration^6^ and the infection with SIV in sergestid shrimp *Acetes erythraeus* with pale and whitish body or strong blue-green iridescence in the severely affected shrimp^5^. Xu *et al*.^8^ did not mention the gross sign except the mortality of freshwater lobster *Cherax quadricarinatus* naturally infected with CQIV. They briefly described cessation of feeding and flaccidity at 5 d post-infection (dpi) followed by a high mortality of freshwater lobster *Cherax quadricarinatus* and *L. vannamei* via intramuscular injection with purified CQIV.

As *mcp* is a highly conserved gene of *Iridoviridae* ^1^, the phylogenetic tree based on the alignment of the complete amino acid sequence of MCP showed that *Chloriridovirus* was located in the branch of *Iridovirus*. It is suggested that it is suitable to use the complete sequence of MCP to evaluate the phylogenetic relationship of different genera of *Iridoviridae*. To examine the phylogenetic relationship of SHIV with other members of family *Iridoviridae*, the amino acid sequences of both MCP and ATPase of SHIV were used in alignments with those of other iridescent viruses of *Iridoviridae* from GenBank. The concatenated phylogeny based on the amino acid sequences of MCP and ATPase showed that the genus *Iridoviridae* was clearly divided into different branches. This concatenated phylogeny may provide a simplified method for analyzing the phylogenetic relationship for *Iridoviridae* rather than use of 26 core iridoviuse genes^1,20^. BLASTX analysis of the MCP showed that SHIV shared 56% identity with that of SIV^5^, which is 1% higher than the comparative result between SIV and CQIV^8^. As no sequence of ATPase of SIV has been published, the phylogenetic tree shown in Fig. 2 did not include SIV, which was located in the branch of genus *Iridovirus* in the phylogenetic tree of MCP. We further compared the MCP sequence of SHIV with the 145 bp MCP sequence of CQIV, which was only reported by Xu *et al*.^8^. It is noteworthy that there is a difference in only one nucleotide in the 145 bp MCP sequences between SHIV and CQIV, indicating that SHIV may be closely related to CQIV. For the reported putative iridovirus in penaeid shrimp *Protrachypene precipua*, its relationship with SHIV cannot be determined, due to the unavailability of the genetic sequence data.

TEM of ultrathin section showed that the size of SHIV particles was larger than those of SIV with diameter of 140 nm^5^ and CQIV with diameter of about 150 nm^8^ and that the diameter of nucleoid (85.8 ± 6.0 nm) was consistent with that of the putative iridovirus from the penaeid shrimp *Protrachypene precipua*, which has a spherically central core or nucleoid with diameter of about 85 nm^6^. SHIV, CQIV, SIV and the putative iridovirus from the *Protrachypene precipua* share the same typical non-enveloped icosahedral structure and they all showed paracrystalline arrays with particle viruses.

Histological examination showed the existence of basophilic inclusions and karyopyknosis in the hematopoietic tissue and hemocytes in gills, hepatopancreas and periopods. Completely assembled virions can also be purified directly from the hemolymph of infected shrimp. Xu *et al*.^8^ also reported that CQIV could be observed in hematopoietic tissue and gills of the infected lobster. The basophilic inclusions were present in the cytoplasmic area in hemocytes or hematopoietic cells of the SHIV-infected shrimp, which is very similar to some reported cases caused by the putative iridovirus in penaeid shrimp *Protrachypene precipua* ^6^ and SIV^5^. The karyopyknosis is similar to some fish cases caused by grouper sleepy disease iridovirus (GSDIV)^21^ and iridovirus in African lampeye and dwarf gourami^22^, but was not reported in shrimp yet. A 279 bp digoxigenin-labeled probe was used to detect SHIV by *in situ* hybridization in sections of infected *L. vannamei*. The probe reacted to the histological lesions in hematopoietic tissue and hemocytes in gills, hepatopancreas and periopods obtained from sample 20141215, as well as in tissues from the challenged experiment described in this study.

SHIV was firstly detected and identified in samples of the diseased shrimp collected from a farm in Zhejiang Province, China, in December 2014. However, results of epidemiological survey indicated that this virus might not be the first outbreak in the farm. Of a total of 89 out of 575 *L. vannamei* individuals, 5 out of 33 *F. chinensis* individuals and 5 out of 10 Mb*. rosenbergii* individuals were SHIV positive in samples collected during 2014–2016 in 20 counties of 5 provinces over China, raising the concern that the virus may have widely spread and aggravated to the surrounding shrimp farming areas.

In conclusion, through isolation, reinfection and histopathological characterization, we have revealed that SHIV is a new virus in family *Iridoviridae* and a pathogen of *L. vannamei*. Additionally, we developed an ISH assay and a nested PCR method for the specific detection of SHIV. These findings underscore the need for professionals versed in aquatic animal health and farmers in the shrimp aquaculture industries to pay closer attention to SHIV and to take more effective measures for preventing the disease outbreaks and economic losses caused by SHIV.

## Materials and Methods

All the protocols of animal handling and sampling were approved by the Animal Care and Ethics Committee, Yellow Sea Fisheries Research Institute, Chinese Academy of Fishery Sciences, and all efforts were made to minimize the suffering of animals according to recommendations proposed by the European Commission (1997). The study was carried out in accordance with the approved protocol. All the methods were applied in accordance with relevant guidelines.

### Shrimp

*Samples of shrimp Litopenaeus vannamei* (No. 20141215) were collected from a farmed pond with high mortality in a farm in Taizhou of Zhejiang Province in December 2014 for viral isolation and metagenomic analysis. For nested PCR tests, *L. vannamei, Fenneropenaeus chinensis, Macrobrachium rosenbergii*, and *Marsupenaeus japonicas* were collected from shrimp farms in the coastal provinces of China during 2014 and 2015. Additionally, healthy juveniles of *L. vannamei* (mean body weight, 3 g) for challenge test were purchased from a shrimp farm in Weifang of Shandong Province, China. The shrimp samples were demonstrated to be free of WSSV, IHHNV, VPAHPND, YHV and TSV by PCR or RT-PCR methods recommended by the World Organization for Animal Health^11,12^.

### Histopathological sections

Samples of the cephalothoraxes were fixed in Davidson’s alcohol formalin acetic acid fixative^13^ for 24 h and then changed to 70% ethanol. Paraffin sections were prepared and stained with H& E staining according to the procedures of Bell & Lightner^13^.

### Construction of a DNA library and sequencing

Sampled cephalothoraxes were homogenized in SM buffer (50 mM Tris-HCl, 10 mM MgSO_4_, 100 mM NaCl, pH 7.5, and 0.5 mM 4-(2-Aminoethyl) benzenesulfonyl fluoride hydrochloride (AEBSF) was added just before use and centrifuged at 10,000 × g for 30 min at 4 °C. The pellet was homogenized in SM buffer and centrifuged at 8,000 × g for 20 min at 4 °C. Homogenization of the pellet in SM buffer and centrifuged at 6,000 × g for 15 min at 4 °C (high speed centrifuge CR21GIII; Hitachi) was repeated. The supernatants of the above steps were merged and filtered through a 0.22 μm membrane and further centrifuged through a 20% (w/w) sucrose cushion at 160,000 × g for 5 h (Ultracentrifuge CP100WX; Hitachi) to precipitate viral particles.

The concentrated viral particles were treated with Turbo DNase (Ambion, Foster City, CA, USA), Benzonase Nuclease (Novagen, Madison, WI, USA) and RNase I (TaKaRa, Dalian, Liaoning, China) to digest the unprotected nucleic acid at 37 °C for 60 min. Viral nucleic acids were extracted using the QIAamp viral DNA kit (Qiagen, Limburg, Netherlands) by following the manufacturer’s instructions.

Viral nucleic acid libraries were constructed by sequence-independent, single-primer amplification (SISPA) PCR amplification. An anchor random primer FR26RV-N (GCCGGAGCTCTGCAGATATCNNNNNN) was used in a reverse transcription reaction with SuperScript III Reverse Transcriptase (Invitrogen, Carlsbad, CA, USA). A single round of DNA synthesis was then performed using Klenow DNA Polymerase (New England BioLab, Ipswich, MA, USA), followed by PCR amplification of nucleic acids using primers consisting of only the 20-base fixed portion of the random primer FR20RV (GCCGGAGCTCTGCAGATATC) with Phusion High-Fidelity DNA Polymerase (Thermo Fisher, USA).

The amplified products were separated by agarose gel electrophoresis and then purified with a QIAquick PCR Purification Kit (Qiagen, Hilden, Germany). High-throughput sequencing was conducted by Novogene (Beijing, China) with the Hiseq. 2000 platform (Illumina, San Diego, CA, US).

### Assembling and phylogenetic analysis

The reads identical to the sequence from the family *Iridoviridae* were assembled using SOAP denovo 2.21 (http://soap.genomics.org.cn/soapdenovo.html). MCP and ATPase sequences were acquired through reads assembling and further analyzed using the tools available at National Center for Biotechnology Information (NCBI) (BLASTx and ORF finder) (http://www.ncbi.nlm.nih.gov). A total of 18 MCP sequences (Table 1) and 14 ATPase sequences (Table 2) were aligned with CLUSTAL_W as implemented in Geneious 9.1.4 using the default settings. A phylogenetic tree was then reconstructed by the maximum likelihood method using Geneious 9.1.4.

### Virus isolation and challenge test

The samples of cephalothoraxes were homogenized in PPB-Tris buffer (376.07 mM NaCl, 6.32 mM K_2_SO_4_, 6.4 mM MgSO_4_, 14.41 mM CaCl_2_, and 50 mM Tris-HCl, pH 6.5–8.0)^23^ and clarified at 9100 × g for 10 min to gather the supernatant. The pellet was homogenized in PPB-Tris buffer again and clarified at 9100 × g for 5 min. Step 3 was repeated three times and the supernatants were combined every time. The supernatant was further centrifuged through a 30% (w/w) sucrose cushion at 36300 × g for 2 h (high speed centrifuge CR21GIII; Hitachi). PPB-Tris buffer was used to suspend the pellet.

Healthy *L. vannamei* shrimp (6 cm in mean length) were cultured temporarily in indoor tanks for 7 days before they were used for challenge tests. For the challenge study, the shrimp were divided into an injection test, a reverse garvage test and a *per os* test. Each test included a virus challenge group and a control group and each group included three biological replicates (20 individuals each). For injection and reverse gavage tests, the crude extracts of SHIV prepared as described above were diluted 100 times with PPB-His buffer, and then every challenge individuals were injected with 15 μL and 200 μL for reverse gavage each individuals in challenge groups. The control groups were disposed with the equal volume of PPB-His buffer. For *per os* infection, shrimp in challenge groups were fed with minced SHIV-infected tissues (5 mm^3^) at 5% of total body weight after 24 h starvation. Thereafter, the shrimp were maintained with pellet feeds for 15 days. At the end of the bioassay, shrimp samples were collected and used for nested PCR and histopathological analysis.

### Probe labeling and ISH

A pair of primers designed from the MCP region was used to amplify the 279 bp fragment using PCR method. PCR reaction mixture (50 μL) contained 1 μL of DNA isolated from SHIV-infected shrimp, 25 μL of I-5^TM^ 2× High Fidelity Master Mix, 0.4 μM each primers (SHIV-ISH-F: 5′-GACCCGAAACTTTATGCA-3′ and SHIV-ISH-R: 5′-CACTTGACACCACCGACT-3′). The PCR was performed at 98 °C for 2 min, followed by 35 cycles of 98 °C for 15 s, 50 °C for 15 s and 72 °C for 10 s, ending with 72 °C for 2 min. Then, the PCR amplified product was separated and analyzed in a 1% agarose gel containing GeneFinder (Bio-V) and 279 bp fragments were excised and purified using the Gel Extraction Kit (Omega). The purified fragments were then used as template to generate the digoxigenin-labeled probe with DIG-High Prime, which was carried out according to the protocol supported by the DIG High Prime DNA Labeling and Detection Starter Kit I (Roche).

The paraffin sections were then subjected to *in situ* hybridization assays according to the protocol of ISH^24^. The tissue of healthy shrimp was used for negative control hybridization.

### TEM

Ultrathin sections of the hepatopancreas from infected shrimp and viral preparations were analyzed under TEM. Small pieces of the hepatopancreas in ~1 mm^3^ of the infected shrimp were fixed in TEM fixative (2% paraformaldehyde, 2.5% glutaraldehyde, 160 mM NaCl and 4 mM CaCl_2_ in 200 mM PBS (pH 7.2) for 24 h at 4 °C. Before ultrathin sectioning, the fixed tissues were secondarily fixed with 1% osmium tetroxide for 2 h, then embedded in Spurr’s resin and stained with uranyl acetate and lead citrate. Ultrathin sections were prepared on collodion-coated grids by the Equipment Center of the Medical College of Qingdao University. A suspension of the virus from the isolation was dropped on the collodion-coated grids and then negatively stained with 2% phosphotungstic acid (pH 7.0). All the grids were examined under a JEOL JEM-1200 electron microscope (Jeol Solutions for Innovation, Peabody, MA, USA) operating at 80–100 kV.

### Viral purification

Hemolymph (50 mL) collected from moribund *L. vannamei* as described above was centrifuged at 1000 × g for 5 min to remove hemocytes, and the supernatant was filtered through a 0.22 μm membrane syringe filter to remove bacteria. The supernatant was further centrifuged through a 30% (w/w) sucrose cushion at 36,300 × g for 2 h (high speed centrifuge CR21GIII; Hitachi, Beckman Coulter Life Science, Brea, CA, USA). The pellet was re-suspended in PPB-Tris buffer and loaded onto a discontinuous sucrose gradient comprised of six layers containing 20, 30, 40, 50 and 60% of sucrose, respectively, and then centrifuged at 50,200 × g for 3 h. Fractions of sucrose gradients were suctioned out and measured for their densities and UV optical density (OD) at 280 nm. An aliquot of the band was dropped on a grid and negatively stained with phosphotungstic acid (PTA) for observation under TEM.

### Nested PCR

Total DNA was extracted from haemolymph and cephalothoraxes of shrimp using TIANamp Marine Animals DNA Kit (Tiangen, Beijing, China). Two pairs of primers targeting the region of ATPase were designed using Primer Premier 5.0 (Premier, Canada). The 25 µL of PCR reaction mixture contained 1 µL of template DNA, 2.5 µL of 10× Ex *Taq* Buffer (Mg^2+^ free), 2 mM MgCl_2_, 0.2 mM dNTPs, 0.4 μM primers (SHIV-F1: 5′-GGGCGGGAGATGGTGTTAGAT-3′ and SHIV-R1: 5′-TCGTTTCGGTACGAAGATGTA-3′) and 0.625 U TaKaRa Ex *Taq* DNA polymerase (TaKaRa, Dalian, Liaoning, China). The PCR was performed at 95 °C for 3 min, followed by 35 cycles of 95 °C for 30 s, 59 °C for 30 s and 72 °C for 30 s, ending at 72 °C for 2 min. In the first step, the PCR amplified a 457 bp amplicon. For the second step of the PCR, in addition to the PCR mixtures that were the same as those described above, different templates and primers (SHIV-F2: 5′-CGGGAAACGATTCGTATTGGG-3′ and SHIV-R2: 5′-TTGCTTGATCGGCATCCTTGA-3′) were also included in the reaction mixtures. The amplification was performed with the following cycling parameters: initial denaturation at 95 °C for 3 min, followed by 35 cycles of 95 °C for 30 s, 59 °C for 30 s and 72 °C for 20 s, and a final extension at 72 °C for 2 min. A 129 bp amplicon was amplified by the second step of the PCR. PCR product (5 µL) was separated and analyzed in a 1% agarose gel containing GeneFinder (Bio-V, China).

To test the specificity of the PCR assay, DNA samples prepared from healthy shrimp and shrimp infected with WSSV, IHHNV, HPV, AHPND and EHP, respectively, were used as templates in the nested PCR assay. In order to determine the limit of detection (LOD) of SHIV nested PCR assay, concentration of DNA extracted from the head tissues of SHIV-infected shrimp was measured using NanoDrop 2000c (Thermo Fisher, USA). Then, DNA samples were diluted in a10-fold serial dilution. The diluted DNAs samples were used as templates for the nested PCR amplification.

### Clinical samples test

In order to investigate the prevalence and distribution of SHIV in China, a total of 625 DNA samples were extracted from hepatopancreas and gills of farmed shrimp collected from ponds distributed in seven provinces of China and tested for the presence of SHIV by the nested PCR specific for SHIV.
